# Enhanced Anaerobic Biodegradation of Benzoate Under Sulfate-Reducing Conditions With Conductive Iron-Oxides in Sediment of Pearl River Estuary

**DOI:** 10.3389/fmicb.2019.00374

**Published:** 2019-03-01

**Authors:** Li Zhuang, Ziyang Tang, Jinlian Ma, Zhen Yu, Yueqiang Wang, Jia Tang

**Affiliations:** ^1^Guangdong Key Laboratory of Environmental Pollution and Health, School of Environment, Jinan University, Guangzhou, China; ^2^Guangdong Key Laboratory of Agricultural Environment Pollution Integrated Control, Guangdong Institute of Eco-environmental Science and Technology, Guangzhou, China

**Keywords:** aromatic compounds, benzoate degradation, sulfate-reducing conditions, (semi)conductive iron oxides, direct interspecies electron transfer, microbial analysis

## Abstract

Anaerobic biodegradation of aromatic compounds under sulfate-reducing conditions is important to marine sediments. Sulfate respiration by a single bacterial strain and syntrophic metabolism by a syntrophic bacterial consortium are primary strategies for sulfate-dependent biodegradation of aromatic compounds. The objective of this study was to investigate the potential of conductive iron oxides to facilitate the degradation of aromatic compounds under sulfate-reducing conditions in marine sediments, using benzoate as a model aromatic compound. Here, in anaerobic incubations of sediments from the Pearl River Estuary, the addition of hematite or magnetite (20 mM as Fe atom) enhanced the rates of sulfate-dependent benzoate degradation by 81.8 and 91.5%, respectively, compared with control incubations without iron oxides. Further experiments demonstrated that the rate of sulfate-dependent benzoate degradation accelerated with increased magnetite concentration (5, 10, and 20 mM). The detection of acetate as an intermediate product implied syntrophic benzoate degradation pathway, which was also supported by the abundance of putative acetate- or/and H_2_-utilizing sulfate reducers from microbial community analysis. Microbial reduction of iron oxides under sulfate-reducing conditions only accounted for 2–11% of electrons produced by benzoate oxidation, thus the stimulatory effect of conductive iron oxides on sulfate-dependent benzoate degradation was not mainly due to an increased pool of terminal electron acceptors. The enhanced rates of syntrophic benzoate degradation by the presence of conductive iron oxides probably resulted from the establishment of a direct interspecies electron transfer (DIET) between syntrophic partners. In the presence of magnetite, Bacteroidetes and Desulfobulbaceae with potential function of extracellular electron transfer might be involved in syntrophic benzoate degradation. Results from this study will contribute to the development of new strategies for *in situ* bioremediation of anaerobic sediments contaminated with aromatic compounds, and provide a new perspective for the natural attenuation of aromatic compounds in iron-rich marine sediments.

## Introduction

Aromatic compounds comprise numerous environmental pollutants and their removal often relies on microbial degradation (Cao et al., 2009). Most contaminated subsurface environments are anaerobic, microbial degradation of aromatic compounds, such as BTEX (benzene, toluene, ethylbenzene, and xylene) or PAH (polycyclic aromatic hydrocarbons), have been observed under nitrate-reducing, Fe(III)-reducing, sulfate-reducing, and methanogenic conditions (Fuchs et al., 2011; Ghattas et al., 2017; Muller et al., 2017; Varjani et al., 2017). This might be the main mechanism in the natural attenuation of aromatic compounds in natural environments, and the supplementation of alternative electron acceptors has been considered as an attractive strategy for bioremediation of sites contaminated with aromatic compounds (Perelo, 2010; Ghattas et al., 2017; Hou et al., 2018; Guo et al., 2019).

Marine sediments are a major final sink and reservoir for aromatic compounds in aquatic environments which often derived from oil spills, industrial waste, sewage effluent, and surface runoff. Due to the high sulfate concentrations in marine sediments, biodegradation of aromatic compounds under sulfate-reducing conditions might be the dominant biodegradation pathway in marine environments. In previous studies, the degradation of aromatic hydrocarbons has been inhibited by suppressing the activity of sulfate-reducing microorganisms or depleting the presence of sulfate in sediments (Lovley et al., 1995). Rothermich et al. (2002) demonstrated biodegradation of *in situ* pools of aromatic hydrocarbons in petroleum-contaminated marine sediments under sulfate-reducing conditions, and suggested that microbial sulfate reduction was the main driving force responsible for the self-purification capacity of contaminated harbor sediments.

In marine ecosystems, many isolated sulfate reducers are capable of mineralizing aromatics with sulfate as the final electron acceptor (Davidova et al., 2007; Musat and Widdel, 2008; Ahn et al., 2009; Musat et al., 2009; Meckenstock and Mouttaki, 2011). As a niche adaption, syntrophic aromatics degradation under sulfate-reducing conditions is also widespread in subsurface environments (Jackson et al., 1999; Kleinsteuber et al., 2008, 2012; Herrmann et al., 2010; Rakoczy et al., 2011; Gieg et al., 2014). In a syntrophic metabolism, aromatics-degrading bacteria cooperate with sulfate-reducing bacteria that can promptly consume end products generated from the breakdown of aromatic compounds (Gibson and Harwood, 2002). Recently, (semi)conductive iron oxides, such as magnetite or hematite, have been demonstrated to be capable of stimulating anaerobic syntrophic metabolism (Kato et al., 2012a,b; Viggi et al., 2014; Baek et al., 2015; Li et al., 2015; Liu et al., 2015; Yamada et al., 2015; Zhuang et al., 2015; Tang et al., 2016; Zhang and Lu, 2016; Wang et al., 2018). In these either laboratory cultures or complex environments, (semi)conductive iron oxides have been proposed to stimulate direct interspecies electron transfer (DIET) in syntrophic interactions. To date, iron oxides-mediated DIET has been mostly observed in syntrophic methanogenesis, the potential of iron oxides to facilitate sulfate-dependent syntrophic metabolism remains unexplored, especially for syntrophic oxidation of aromatic compounds.

Benzoate is most frequently used as a model compound for studying anaerobic metabolism of aromatic compounds (Young and Frazer, 1987). Benzoate biodegradation under sulfate-reducing conditions may occur through direct sulfate reduction by a single culture (Drzyzga et al., 1993; Madan and Bernhard, 2015) or, syntrophically, by a consortium of bacteria (Hopkins et al., 1995). The aim of the present study was to investigate the potential of (semi)conductive iron oxides to enhance the rate of benzoate degradation under sulfate-reducing conditions, which might provide effective bioremediation technology for marine sediments contaminated by aromatic compounds. Here, in the absence and presence of magnetite or hematite, the coupling of benzoate degradation and sulfate reduction was analyzed in anaerobic incubations of sediment from the Pearl River Estuary, China. Using 16S rRNA sequencing, microbial communities from sulfate-dependent benzoate degradation in the absence and presence of conductive iron oxides were characterized to further elucidate the microbial syntrophic mechanisms involved.

## Materials and Methods

### Sediment Sample Collection

Sediment samples (0–15 cm) were collected from the Pearl River Estuary crossing Guangzhou (23°6′36″N, 113°18′21″E) in China. This river section is one of the most important ecosystems relating developing land areas to the South China Sea, which is characterized by excessive inputs of anthropogenic contaminants from wastewater discharges. All samples were sieved through 2.0 mm pore size to remove coarse debris and gravel, and then stored in anaerobic packs (AnaeroPack, MGC. CO. Inc, Japan) to maintain anaerobic conditions, at 4°C for 1 month until use. The physio-chemical properties were determined as described by Chen et al. (2016) and the results showed: pH (7.1), total organic carbon (7.3%), total Fe (3.2%), total nitrogen (0.2%), and SO_4_^2-^ (4.8 mg/kg).

### Anaerobic Incubation Experiments

To investigate the effect of (semi)conductive iron oxides on benzoate degradation under sulfate-reducing conditions, a series of anaerobic incubation experiments were conducted (see details in Table 1). Before the anaerobic incubation, the sediment samples were enriched in a modified anaerobic medium (Kato et al., 2010) (1:3, w/v) with the supplementation of 3 mM benzoate and 12 mM sulfate under anaerobic conditions (N_2_:CO_2_ [(80:20) v/v]). The anaerobic medium consisted of (L^-1^) 0.535 g NH_4_Cl, 0.055 g CaCl_2_, 0.047 g MgCl, 0.42 g NaHCO_3_, 2.38 g 4-(2-hydroxyethyl)-1-piperazineethanesulfonic acid (Hepes), 1 mL trace element solution and 10 mL vitamin solution (Li et al., 2015). All the incubations were carried out in 125 mL serum bottles, each containing 5 mL of sediment enrichment, 45 mL of anaerobic medium, 3 mM benzoate, and 12 mM sulfate (if required). Hematite or magnetite was added to a final concentration of 20 mM as Fe atom. Two feeding cycles were conducted for anaerobic incubations with and without iron oxides, and each dose of benzoate and sulfate were about 2.2∼2.4 mM and 11.7∼12.3 mM, respectively. When benzoate was completely degraded, benzoate and sulfate were replenished to the levels similar to those in the first feeding cycle and the second feeding cycle was initiated.

**Table 1:** The details of each anaerobic incubation.

	Treatment	Benzoate	Sulfate	Iron oxide
(Semi) conductive iron oxides	Benzoate	3 mM		
	Benzoate + 20 mM Mag	3 mM		20 mM magnetite
	Benzoate + SO_4_^2-^	3 mM	12 mM	
	Benzoate + SO_4_^2-^ + 20 mM Mag	3 mM	12 mM	20 mM magnetite
	Benzoate + SO_4_^2-^ + 20 mM Hem	3 mM	12 mM	20 mM hematite
NTA-Fe(III) as electron acceptor	Benzoate	3 mM		
	Benzoate + SO_4_^2-^	3 mM	12 mM	
	Benzoate + 10 mM Fe-NTA	3 mM		10 mM Fe(III)-NTA
	Benzoate + SO_4_^2-^ + 10 mM Fe-NTA	3 mM	12 mM	10 mM Fe(III)-NTA
Different concentration of magnetite	Benzoate	3 mM		
	Benzoate + 20 mM Mag	3 mM		20 mM magnetite
	Benzoate + SO_4_^2-^	3 mM	12 mM	
	Benzoate + SO_4_^2-^ + 5 mM Mag	3 mM	12 mM	5 mM magnetite
	Benzoate + SO_4_^2-^ + 10 mM Mag	3 mM	12 mM	10 mM magnetite
	Benzoate + SO_4_^2-^ + 20 mM Mag	3 mM	12 mM	20 mM magnetite


Under the same experimental conditions, the sulfate-free, iron oxides-amended experiments were conducted to test the potential of iron oxides as additional electron acceptors for benzoate oxidation. To test the effect of microbial Fe(III) reduction on benzoate degradation [reaction of sulfide produced by sulfate reduction with Fe(II)], anaerobic incubations with the addition of 10 mM Nitrilotriacetate-Fe(III) [NTA-Fe(III)] were performed. To explore the association between magnetite loading and sulfate-dependent benzoate degradation, incubations with different concentrations of magnetite (5, 10, and 20 mM as Fe atom) were carried out.

The stock solution of NTA-Fe(III) was prepared by mixing 3.9 M NaHCO_3_, 0.19 M Nitrilotriacetic acid, and 0.2 M FeCl_3_ in Milli-Q water (Park et al., 2008). Hematite was synthesized by sintering lepidocrocite powder (prepared by mixing 0.08 M FeCl_2_, 0.16 M (CH_2_)_6_N_4_ and 0.8 M NaNO_2_ in Milli-Q water) at 420°C for 2 h with an incremental increase of 2°C min^-1^ (Li et al., 2007). Magnetite nanoparticles (50∼100 μm) were purchased from Sigma-Aldrich Inc. (Germany). All the incubation bottles were sealed with Teflon^®^-coated septa and aluminum crimp caps after being bubbled with N_2_:CO_2_ [(80:20) v/v] gas at a rate of 10 mL⋅min^-1^ for 1 h. All experiments were performed in biological triplicate and incubated at 30°C in a constant temperature incubator in the dark.

### Chemical Analysis

The concentrations of acetate and benzoate were determined with HPLC (Shimadzu LC-15C, Japan) equipped with a C_18_ reverse-phase column (150 mm by 4.6 mm) and a UV detector. The benzoate was detected at 231 nm with a mobile phase of 78% KH_2_PO_4_ [50 mM, supplemented with 23% (v/v) acetic acid and isopropanol] and 22% methanol at a flow rate of 1.0 mL min^-1^. The detection limit of benzoate was 2 μM. For acetate, the instrument was operated with the UV detector set at 210 nm, a mobile phase of phosphate buffer (18 mM, pH 2.15) at a flow rate of 0.5 mL min^-1^. The detection limit of acetate was 10 μM. Sulfate concentrations were measured by ion chromatography (IC) (Dionex ICS900; Thermo Fisher Scientific, United States) equipped with AERS 500 and with an eluent of carbonate (1.8 mM)-bicarbonate (1.7 mM) at a flow rate of 1.0 mL min^-1^. The HCl-extractable Fe(II) concentrations were determined via the Ferrozin colorimetric method, as described previously (Lovley and Phillips, 1986).

### Microbial Community Analysis

Samples from anaerobic incubations (Benzoate, Benzoate+20 mM magnetite, Benzoate+SO_4_^2-^, Benzoate+SO_4_^2-^+5, 10, or 20 mM magnetite) at the end of the incubation period and the sediment enrichment (D0) used as inocula for anaerobic incubations were sampled for microbial community analysis. DNA from each sample was extracted using Power-Soil^TM^ DNA isolation KIT (Mo Bio Laboratories, United States) according to the manufacturer’s instructions. The concentration and quality of DNA were tested using a NanoDrop spectrophotometer (Thermo Fisher Scientific, United States). The extracted DNA was diluted to 10 ng μL^-1^ and stored at -80°C for downstream use.

The primer set of 515F (5′-GTGCCAGCMGCCGCGGTAA-3′) and 806R (5′-GGACTACHVGGGTWTCTAAT-3′) with 12 nt unique barcodes was used for amplifying the V4 hypervariable regions of the bacterial and archaeal 16S rRNA genes. The PCR mixture (30 μL) contained 0.75 units Ex-Taq DNA polymerase (TaKaRa, Dalian, China), 1 × Ex Taq loading buffer (TaKaRa, Dalian, China), 0.2 mM dNTP mix (TaKaRa, Dalian, China), 0.2 μM of each primer, and 100 ng of template DNA. The PCR amplification program included an initial denaturation at 94°C for 5 min, followed by 35 cycles of 94°C for 30 s, 53°C for 60 s and 72°C for 60 s, and a final elongation at 72°C for 7 min. The PCR products were subjected to electrophoresis using a 1.0% agarose gel. The band with correct size was excised and purified using SanPrep DNA Gel Extraction Kit (Sangon Biotech, China) and quantified with NanoDrop (Thermo Fisher Scientific, United States). All purified amplicons were pooled in equal molar amounts and sequenced using an Illumina Miseq system by Novogene Bioinformatics Technology (Beijing, China).

The sequencing data were processed according to Kozich et al. (2013). Reads shorter than 300 bases and whose barcode and primer did not match as expected were discarded. The trimmed reads were then denoised as described by Caporaso et al. (2012), and chimeric and low-quality sequences were removed using the UCHIME algorithm within MOTHUR (Edgar et al., 2011). The operational taxonomic units (OTUs) were defined at the sequence similarity level of 97% using the QIIME software package (Caporaso et al., 2012), and a representative sequence from each OTU was assigned to a taxonomic identity using the SILVA Database Project with a minimum confidence level of 80% (Quast et al., 2013). The weighted Unifrac distance was calculated for principal coordinate analysis (PCoA). The raw sequence reads were deposited in the sequence read archive (SRA) of the NCBI with accession numbers from SRR8275555 to SRR8275575.

### Statistical Analysis

The significant differences among treatments were detected using the ANOVA function in the SPSS software package (SPSS Inc., 2005). Differences among values were considered to be statistically significant at *P* < 0.05.

## Results

### Enhanced Sulfate-Dependent Benzoate Degradation by the Presence of Iron Oxides

**FIGURE 1:**
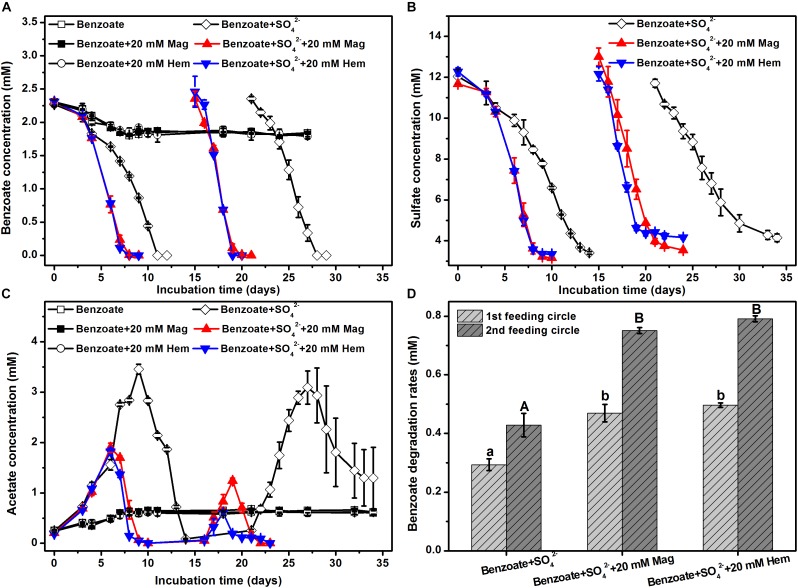
Sulfate-dependent benzoate degradation in the anaerobic incubation of sediments from Pearl River Estuary in the presence and absence of conductive iron oxides: **(A)** benzoate degradation; **(B)** sulfate reduction; **(C)** acetate concentration; **(D)** benzoate degradation rates (calculated from the phase with fastest benzoate degradation). Two feeding cycles were included. The lowercases and capitals letters indicate the significant differences among three treatments in the first and the second feeding cycle, respectively. Mag and Hem denote magnetite and hematite, respectively.

In the control incubations without sulfate and iron oxides, benzoate was initially degraded, but the degrading capability was not sustained (Figure 1A). The addition of magnetite or hematite did not improve benzoate degradation in the absence of sulfate compared to the controls with benzoate addition only, suggesting that both magnetite and hematite were not serving as electron acceptors for benzoate oxidation. However, benzoate degradation was stimulated by the presence of sulfate independent of the addition of iron oxides. During the first feeding cycle, benzoate was completely removed within 8 days in the incubations amended with magnetite or hematite, while it took 11 days for the iron oxides-free incubations to achieve complete degradation (Figure 1A). In the second feeding cycle, the time periods for complete removal of benzoate in the incubations with and without iron oxides were shortened to 4 and 7 days, respectively. Sulfate reduction occurred simultaneously with anaerobic degradation of benzoate, and the depletion rates in the iron oxides-amended incubations were faster than that in the iron oxides-free incubations for both the first and the second feeding cycles (Figure 1B). During the anaerobic, sulfate-dependent benzoate degradation, acetate was detected as an intermediate product that exhibited a pattern of gradual increase to a maximum, followed by a decrease in concentration (Figure 1C). In the first feeding cycle, the maximum concentrations of acetate were 1.8–1.9 and 3.4 mM for the incubations with and without iron oxides, respectively. For the iron oxides-amended incubations, the maximum concentration of acetate in the second feeding cycle was lower than that in the first feeding cycle, and the time period for complete acetate degradation in the second feeding cycle became shorter (6 days). Based on the data during the linear phase of benzoate metabolism (Figure 1A), the degradation rates of benzoate in all incubations were estimated (Figure 1D). In the first feeding cycle, benzoate degradation rates were increased from 0.27 to 0.46 or 0.50 mM/d by the supplementation of magnetite or hematite, respectively. In the second feeding cycles, the rates of sulfate-depended benzoate degradation became faster than those in the first feeding cycle for all incubations. More importantly, the enhancement of degradation rates by iron oxides showed a greater extent compared with the first feeding cycle (81.8% increased by magnetite, 91.5% increased by hematite).

For all incubations with sulfate-dependent benzoate degradation, acetate transiently accumulated and was further metabolized. In which case, each mole of benzoate oxidized could result in 3.75 mol of sulfate being reduced according to Equation 1. In the presence of sulfate, microbial reduction of magnetite and hematite occurred (Supplementary Figure S1), they might act as the terminal electron acceptor for benzoate oxidation according to Equation 2 (Lovley et al., 1989).

(1) $$C_{7}H_{5}O_{2}{}^{-}+3.75SO_{4}{}^{2-}+4H_{2}O\to 7HCO_{3}{}^{-}+3.75HS^{-}+2.25H^{+}$$

(2) $$C_{7}H_{5}O_{2}{}^{-}+30Fe(III)(s)+19H_{2}O\to 30Fe(II)(l)+7HCO_{3}{}^{-}+36H^{+}$$

In all sediment incubations, sulfate reduction accounted for 85–107% of electrons expected from benzoate oxidation (Table 2), while Fe(III) reduction only accounted for 2–11% of electrons produced by benzoate oxidation (Supplementary Table S1). This suggests that sulfate was the predominant terminal electron acceptor during the anaerobic degradation of benzoate.

**Table 2:** Stoichiometry and electron balance during anaerobic, sulfate-dependent benzoate degradation.

Treatment	Feedingcycle	Benozoate degraded (mM)	Electrons produced from benzoate oxidation (mmol)	Sulfate depleted (mmol)	Electrons consumed by sulfate reduction (mmol)	Fe(II) reduced (mmol)	Electrons consumed by Fe(III) reduction (mmol)	Total electron recovery
Iron oxides-free	1st	2.27	68.1	8.63	69.0	/	/	101%
	2nd	2.36	70.8	7.56	60.5	/	/	85%
Magnetite-amended	1st	2.32	69.6	8.51	68.1	7.5	7.5	109%
	2nd	2.36	70.8	9.46	75.7	1.6	1.6	109%
Hematite-amended	1st	2.31	69.3	8.92	71.4	7.0	7.0	113%
	2nd	2.46	73.8	8.00	64.0	1.6	1.6	89%


**FIGURE 2:**
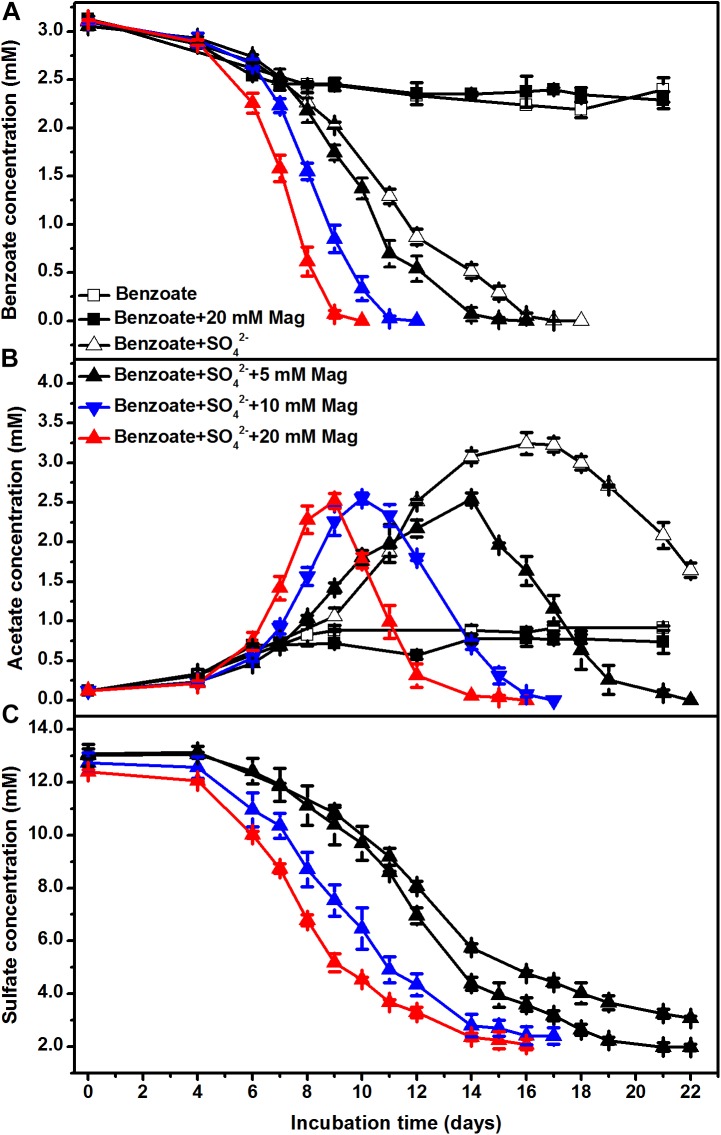
The effect of magnetite loading on sulfate-dependent benzoate degradation: **(A)** benzoate degradation; **(B)** acetate concentration; **(C)** sulfate reduction. The concentrations of magnetite were tested at 5, 10, and 20 mM. Mag denotes magnetite.

To further investigate the stimulatory effect of iron oxides on sulfate-dependent benzoate degradation, we applied different concentrations of magnetite (5, 10, and 20 mM) to the sediment incubations. Under sulfate-reducing conditions, benzoate added at an initial concentration of ca. 3 mM completely disappeared after 18, 16, 12, and 10 days of incubation in the presence of 0, 5, 10, and 20 mM magnetite (Figure 2A), respectively. The rates of benzoate degradation and sulfate reduction (Figure 2C) accelerated with increased addition of magnetite, as well as the degradation of acetate (Figure 2B).

### The Effect of NTA-Fe(III) on Sulfate-Dependent Benzoate Degradation

**FIGURE 3:**
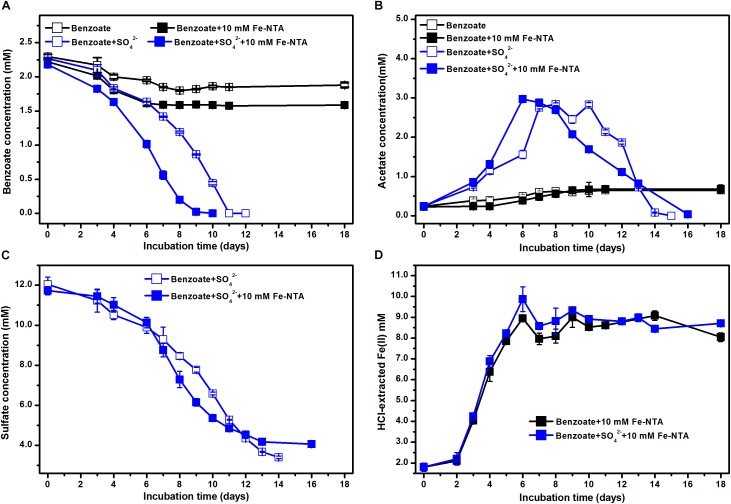
Effect of NTA-Fe(III) addition on benzoate degradation under sulfate-reducing conditions: **(A)** benzoate degradation; **(B)** sulfate reduction; **(C)** acetate concentration; **(D)** concentrations of Fe(II). Fe-NTA denotes ferric nitrilotriacetate.

In the absence of sulfate, the incubations with NTA-Fe(III) degraded 0.22 mM benzoate more than that in the incubations without NTA-Fe(III). Benzoate was completely removed under sulfate-reducing conditions within 10 and 11 days in the presence and the absence of NTA-Fe(III), respectively (Figure 3A). Similarly, acetate was accumulated and further degraded completely after 15 and 16 days of sulfate-dependent benzoate degradation in the absence and presence of NTA-Fe(III), respectively (Figure 3B). Fe(III) was mainly reduced during the early incubation period, producing 8.1 and 7.2 mM Fe(II) in the benzoate-fed incubations with and without sulfate, respectively (Figure 3D). In the incubations with NTA-Fe(III), the sum of sulfate reduction (Figure 3C) and Fe(III) reduction was responsible for 103% of electron expected from benzoate oxidation.

**FIGURE 4:**
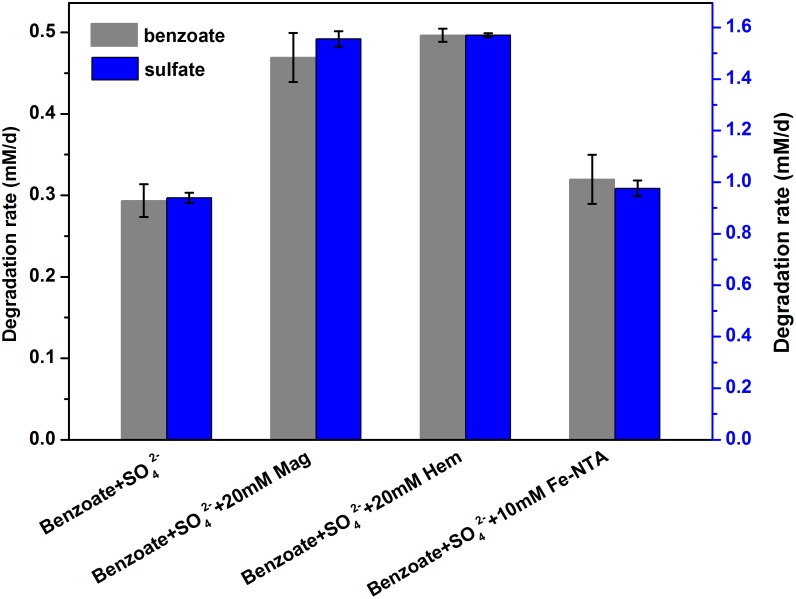
Comparison of the rates of benzoate degradation and sulfate reduction with different iron oxides [NTA-Fe(III), magnetite and hematite]. The different lowercase letters denote significant difference between the four treatments. Mag, Hem, and Fe-NTA denote magnetite, hematite and ferric nitrilotriacetate, respectively.

Estimated from the linear phase of benzoate degradation kinetics (Figure 3A), the rates of sulfate-dependent benzoate degradation in the presence of NTA-Fe(III) (0.32 mM/d) was comparable to that without NTA-Fe(III) (0.29 mM/d), which was significantly lower than the rates of sulfate-dependent benzoate degradation with the supplementation of magnetite (0.47 mM/d) or hematite (0.50 mM/d) (Figure 4). A similar trend was found for sulfate reduction. These results suggest that the facilitating effect of magnetite or hematite was not primarily due to microbial Fe(III) reduction, acting as an terminal electron acceptor for benzoate oxidation.

### Microbial Community Analysis

**FIGURE 5:**
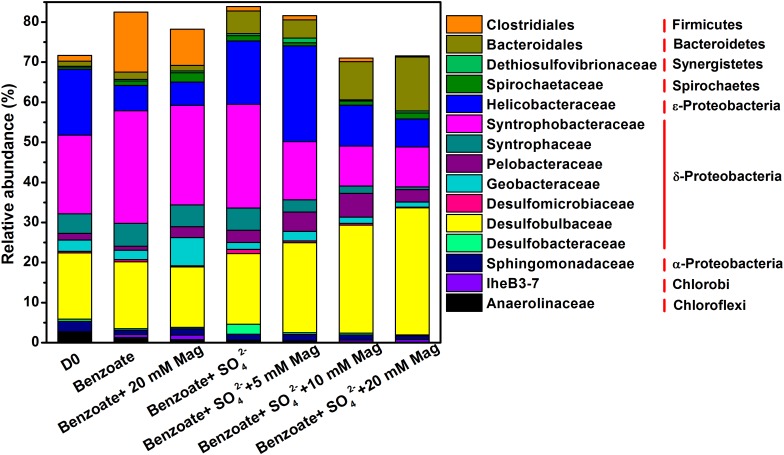
Taxonomic classification of 16S rRNA gene sequences from the extract DNA from anaerobic incubation of sulfate-dependent benzoate degradation in the presence and absence of magnetite, the sulfate-free control incubations and microbial inocula at the family level with a relative abundance greater than 1%. Mag denotes magnetite.

An average of 54091 quality sequences (ranging from 47086 to 62895) and an average of 1973 OTUs (ranging from 960 to 2803) per sample remained following sequencing processing. 16S rRNA-based microbial community composition according to these data is shown in Figure 5, which included the dominant phylotypes at the family level (with a relative abundance greater than 1%). For all sediment samples, the most abundant bacteria belonged to the δ-Proteobacteria order, accounting for 46.5–57.4% of the total quality sequences in all libraries. Within the δ-Proteobacteria, the families of Syntrophobacteraceae and Desulfobulbaceae were the most predominant phylotypes. Compared with the incubations without magnetite, a decreasing trend of Syntrophobacteraceae and an increasing trend of Desulfobulbaceae with increasing concentrations of magnetite were observed. Helicobacteraceae was the prominent phylotype in the ε-Proteobacteria order with a proportion of 6.3–23.9%, and its presence was highly enriched in the incubations of benzoate+SO_4_^2-^+5 mM magnetite. Fermentative Clostridiales was predominant only in the sulfate-free control incubations (9.0–15.0%), and the relative abundance decreased dramatically in the incubations of sulfate-dependent benzoate degradation (0.3–1.1%). Relative to the low abundance in the sulfate-free incubations (1.4–1.9%), members of the Bacteroidales family were enriched by benzoate degradation under sulfate-reducing conditions, and the amounts of enrichment increased with increasing magnetite concentration (4.5, 9.5, and 13.4% for 5, 10, and 20 mM magnetite-amended incubations, respectively). For sulfate-dependent benzoate degradation without magnetite, the proportions of the families of both Desulfomicrobiaceae and Desulfobacteraceae were 1.0 and 2.5% respectively, but they decreased with magnetite amendments. In the incubations of benzoate+SO_4_^2-^+20 mM magnetite, the abundances of Desulfomicrobiaceae and Desulfobacteraceae decreased to 0.1 and 0.2%, respectively.

**FIGURE 6:**
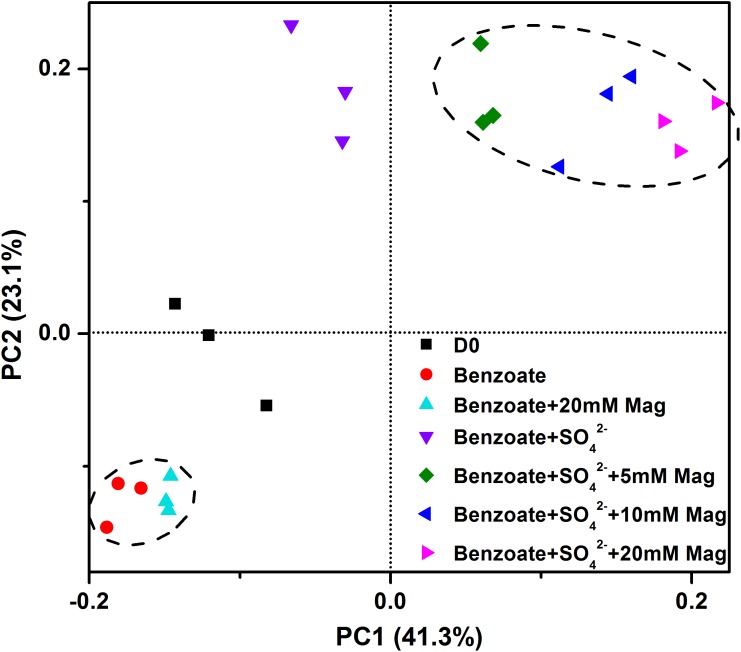
Principal coordinates analysis (PCoA) of microbial communities from sediments incubations of sulfate-dependent benzoate degradation in the presence and absence of magnetite, the sulfate-free control incubations and microbial inocula based on the weighed unifrac distance. Mag denotes magnetite.

Figure 6 shows the clustering of these microbial communities by weighted fast UniFrac PCoA. Microbial communities from sulfate-dependent benzoate degradation in the presence of magnetite formed a distinct cluster, which was separated from that of sulfate-dependent benzoate degradation without magnetite by the first principal component (PC1). Communities from benzoate degradation without sulfate were grouped together, and discriminated from that of sulfate-dependent benzoate degradation by the second principal component axis (PC2). These vectors illustrate that the addition of magnetite discriminated communities on PC1, and the presence of sulfate as a terminal electron acceptor for benzoate degradation discriminated communities on PC2.

## Discussion

### Syntrophic Benzoate Degradation Under Sulfate-Reducing Conditions

Under sulfate-reducing conditions, benzoate can be oxidized completely to CO_2_ (Equation 1); free hydrogen or acetate is not expected in this metabolism (Drzyzga et al., 1993). However, acetate was detected during sulfate-dependent benzoate degradation in the present study, which suggests the occurrence of incomplete oxidation of benzoate. There are two pathways of sulfate-dependent benzoate degradation with the appearance of acetate as an intermediate product: (i) using sulfate as electron acceptor, sulfate reducers oxidize benzoate incompletely to produce acetate (Equation 3), which might be further utilized by other acetate-utilizing sulfate reducers (Equation 4) (Kamagata et al., 1992); (ii) benzoate is syntrophically degraded by acetate/H_2_-producing benzoate-degraders and acetate/H_2_-using sulfate reducers (Equations 5–7) (Fang et al., 1997).

(3) $$C_{7}H_{5}O_{2}{}^{-}+0.75SO_{4}{}^{2-}+4H_{2}O\to 3CH_{3}COO^{-}+HCO_{3}{}^{-}+0.75HS^{-}+2.25H^{+}\Delta {G^{\prime }}_{0}=-81.4kJ/reaction$$

(4) $$CH_{3}COO^{-}+SO_{4}{}^{2-}\to 2HCO_{3}{}^{-}+HS^{-}\Delta {G^{\prime }}_{0}=-47.6kJ/reaction$$

(5) $$C_{7}H_{5}O_{2}{}^{-}+7H_{2}O\to 3H_{2}+HCO_{3}{}^{-}+3CH_{3}COO^{-}+3H^{+}\Delta {G^{\prime }}_{0}=+70.5kJ/reaction$$

(6) $$CH_{3}COO^{-}+SO_{4}{}^{2-}\to 2HCO_{3}{}^{-}+HS^{-}\Delta {G^{\prime }}_{0}=-47.6kJ/reaction$$

(7) $$4H_{2}+SO_{4}{}^{2-}+H^{+}\to HS^{-}+4H_{2}\mathrm{O\Delta}{G^{\prime }}_{0}=-151.9kJ/reaction$$

Although the incomplete oxidization of benzoate to acetate coupling sulfate reduction is thermodynamically favorable (Equation 3), to date, not a single sulfate-reducing microorganism has been reported to be responsible for this incomplete oxidation of benzoate under sulfate-reducing conditions. Kamagata et al. (1992) found that an anaerobic consortium degraded benzoate producing acetate with a sulfate reducer that did not oxidize benzoate, which implied that syntrophic interaction was essential for benzoate degradation. For the second pathway, the need for syntrophic metabolism is determined by the energetically unfavorable reaction of benzoate oxidation to acetate (Equation 5), which can become exergonic if sulfate reducers keep acetate or H_2_ at very low concentrations by active consumption (Equations 6–7). In the present study, based on the presence of acetate during sulfate-dependent benzoate degradation, syntrophic metabolism was suggested to be highly involved.

In all anaerobic incubations of sediment, the most predominant bacterial sequences were related to δ-Proteobacteria which include all gram-negative mesophilic sulfate-reducing bacteria. The known sulfate-reducing bacteria observed in the present study were Desulfobulbaceae (15.0–31.7%), Syntrophobacteraceae (10.0–28.1%), Desulfobacteraceae (0.21–2.5%), and Desulfomicrobiaceae (0.13–1.0%). These sulfate-reducing taxa implied that they were potentially responsible for sulfate reduction during benzoate degradation in the present study. The family Desulfobulbaceae comprises physiologically versatile sulfate reducers, and the cultured representatives are capable of utilizing H_2_, acetate, propionate, lactate, pyruvate, and alcohols (Kuever, 2014a; Rabus et al., 2015). Recently, a species of *Desulfoprunum benzoelyticum* within the family Desulfobulbaceae has been identified to be capable of utilizing benzoate with sulfate reduction (Madan and Bernhard, 2015), however, the metabolism is complete oxidation without acetate production. Syntrophobacteraceae are the second most important sulfate reducers detected in this experiment. Majority of them are able to use H_2_ or acetate to reduce sulfate, but their capability of utilizing benzoate for sulfate reduction has not been reported. Syntrophobacteraceae affiliated species have been identified as the major acetate-degrading sulfate reducers in Italian paddy soil (Liu et al., 2018). Members within the family Desulfobacteraceae are either mesophilic or psychrophilic sulfate-reducing bacteria, and some of them can completely oxidize benzoate under sulfate-reducing conditions. Although, all members within the Desulfomicrobiaceae family can oxidize organic substrates incompletely to acetate with sulfate reduction, benzoate is not included (Kuever and Galushko, 2014). In summary, the phylotypes affiliated to sulfate-reducing δ-Proteobacteria in this experiment were mainly putative acetate- or/and H_2_-utilizing sulfate reducers.

Bacteria capable of syntrophic metabolism largely belong to δ-Proteobacteria (Mcinerney et al., 2008), here we detected the well-known syntrophic bacteria including Syntrophobacteraceae, Syntrophaceae, and Geobacteraceae with a relative abundance of > 1%. Among them, species of Syntrophaceae generally oxidize benzoate incompletely with H_2_-utilizing partners (Kuever, 2014b). For example, Syntrophus species have been reported to grow syntrophically on benzoate with H_2_-using sulfate reducers (Auburger and Winter, 1995; Hopkins et al., 1995; Elshahed et al., 2001). All Geobacteraceae can oxidize acetate (Röling, 2014), and several species are capable of degrading benzoate (Coates et al., 2001). Species affiliated to the family Geobacteraceae do not use sulfate as an electron acceptor (Röling, 2014), but can act as syntrophic acetate oxidizer in the presence of hydrogenotrophic partner (Kato et al., 2012b). Syntrophobacteraceae are capable of fermentative metabolism or growing in syntrophic association with H_2_/formate-utilizing partner (Kuever, 2014c). Thus, the presence of various syntrophic bacteria suggested their role to oxidize benzoate or acetate with the production of H_2_, and potentially live associated with hydrogenotrophic sulfate-reducing bacteria.

In the present study, the detection of acetate during sulfate-dependent benzoate degradation, as well as microbial communities that characterized with the abundant presence of syntrophic benzoate/acetate oxidizer and acetate/H_2_-utilizing sulfate reducers demonstrated that benzoate was not degraded by a single sulfate reducer, but by syntrophic metabolism of several different microorganisms.

### Conductive Iron Oxides Accelerated Syntrophic Benzoate Degradation

In the absence of sulfate, microbial reduction of magnetite or hematite was insignificant, and they were not serving as electron acceptors for benzoate oxidation (Figure 1A). In the presence of sulfate, both magnetite and hematite reduction were significantly enhanced, which was consistent with previous findings that iron reduction can be enhanced during bacterial sulfate reduction (Li et al., 2006). Fe(II) production from magnetite or hematite reduction in the presence of sulfate was close to (first feeding cycle) or much lower than (second feeding cycle) that from NTA-Fe(III) reduction. Thus, the very comparable rates of sulfate-dependent benzoate degradation in the absence and presence of NTA-Fe(III) (Figure 4) might help to eliminate two possibilities for the stimulatory effect of conductive iron oxides on benzoate degradation under sulfate-reducing conditions: (i) the presence of iron oxides increasing the pool of terminal electron acceptors; (ii) decreasing H_2_S toxicity by FeS formation via Fe(II) and sulfide (caused by sulfate reduction).

Conductive iron oxides have been documented to accelerate a range of anaerobic syntrophic interactions, including methanogenesis (Kato et al., 2012b; Viggi et al., 2014; Li et al., 2015; Yamada et al., 2015; Zhuang et al., 2015), dechlorination (Aulenta et al., 2013, 2014) and nitrate reduction (Kato et al., 2012a). It has been proposed that conductive minerals have stimulatory effect by promoting DIET between electron-donating microorganisms and electron-accepting microorganisms responsible for syntrophic metabolism. The function of conductive minerals has been suggested as an electron conduit for DIET (Kato et al., 2012b) or a substitute for pilin-associated c-type cytochrome (Liu et al., 2015). As an alternative to traditional interspecies H_2_/formate transfer, the interspecies electron transfer occurred via electrical conduction is faster than Fick’s law-based H_2_ diffusion, making DIET advantageous over interspecies H_2_/formate transfer. In the present study, since syntrophic interactions were necessary for benzoate degradation under sulfate-reducing conditions, the facilitated benzoate degradation by the addition of conductive iron oxides was likely due to the establishment of DIET between syntrophic benzoate oxidizers and sulfate reducers.

As the rates of sulfate-dependent benzoate degradation increased with increasing addition of magnetite, the relative abundances of Bacteroidetes and Desulfobulbaceae also showed an increasing trend with the increasing dose of magnetite. Although, the physiological function of Bacteroidales in oxidizing benzoate has not been reported so far, species of Bacteroidales are known to be capable of anaerobic degradation of complex substrates to simple sugars and other products (Xu et al., 2003). Dawson et al. (2012) observed dense aggregates of a methanogenic community and a bacterial community consisting of *Acetobacterium* spp., Bacteroidales, and SRB385-hybridizing Firmicutes, which were responsible for syntrophic interactions in a biogenic gas field containing aromatic compounds. In a benzene-contaminated aquifer with ongoing sulfate reduction, Bacteroidales has been identified as the dominant order of bacteria (Herrmann et al., 2008). These findings provide evidence for the involvement of Bacteroidales in aromatic compounds degradation under anaerobic conditions.

To participate in DIET, microorganisms must be capable of extracellular electron transfer. Bacteroidetes have been detected as the predominant microorganisms in the anode biofilm in microbial fuel cells (MFC) fed with complex organic compounds (Kim et al., 2006; Zhang et al., 2009) and their function of extracellular electron transfer were evidenced by Fe(III)-reducing activity and electrochemical activities (Kim et al., 2006). The family Desulfobulbaceae was found to consistently be enriched on the surface of electrodes generating electricity from marine sediment fuel cells (Tender et al., 2002; Holmes et al., 2004b). For example, *Desulfobulbus propionicus* was the first sulfate-reducing bacteria capable of conserving energy to support growth via electron transfer to insoluble electron acceptors (iron oxides and electrodes) (Holmes et al., 2004a). Considering that both Bacteroidetes and Desulfobulbaceae have the potential capability for extracellular electron transfer, as well as their enrichment with magnetite supplementation, they might be involved in magnetite-mediated DIET during the course of benzoate degradation coupled with sulfate reduction. Most probably, in the presence of magnetite, electrons generated from the metabolic oxidation of benzoate by Bacteroidetes were transferred via electrical conduction to Desulfobulbaceae for reducing sulfate (Supplementary Figure S2). However, this hypothesis warrants further investigations.

In the present study, sulfate-dependent benzoate degradation produced acetate as the primary intermediate that was further oxidized. Though acetate oxidation coupled to sulfate reduction can be performed by a single microorganism, the relative abundances of Syntrophobacteraceae that are capable of acetate-consuming sulfate reduction decreased with the increasing concentration of magnetite. In comparison, the abundances of Desulfobulbaceae that comprises many genera capable of utilizing H_2_ as an electron donor were increased with the increasing loading of magnetite. As well as being performed by a single organism, sulfate-dependent acetate oxidation might also occur through syntrophic associations according to the following equations (Equations 8 and 9).

(8) $$CH_{3}COOH+2H_{2}O\to 4H_{2}+2CO_{2}\Delta {G^{\prime }}_{0}=+104.6kJ/reaction$$

(9) $$4H_{2}+SO_{4}{}^{2-}+H^{+}\to HS^{-}+4H_{2}\mathrm{O\Delta}{G^{\prime }}_{0}=-151.9kJ/reaction$$

Magnetite-facilitated syntrophic acetate oxidation has been reported under methanogenic conditions, in which magnetite stimulated DIET between *Geobacter* and *Methanosarcina* species (Kato et al., 2012b; Zhou et al., 2014; Rotaru et al., 2018). The possibility of magnetite-stimulated DIET between acetate-oxidizing bacteria and H_2_-using sulfate reducer (Supplementary Figure S3) can be further evidenced by the co-culture of *Geobacter sulfurreducens* (oxidizes acetate but cannot use sulfate) and *Desulfovibrio* sp. (reduces sulfate but cannot use acetate).

## Conclusion

In summary, using sediment from Pearl River Estuary as microbial inocula, the present study demonstrated that the supplementation of (semi)conductive iron oxides, magnetite or hematite, accelerated the rate of benzoate degradation under sulfate-reducing conditions. The detection of acetate, along with microbial analysis implied that syntrophic dependence was essential to benzoate degradation under sulfate-reducing conditions. The stimulatory effect of (semi)conductive iron oxides on sulfate-dependent benzoate degradation could be a result of stimulating DIET within syntrophic microorganisms. This hypothesis, however, warrants further investigation. An increasing number of studies have demonstrated the capability of conductive iron oxides to promote DIET, which highlights the potential application of iron oxides in industrial and environmental biotechnologies such as bioremediation. Considering the ubiquity of conductive iron oxides in soils and sediments, DIET-facilitated syntrophic metabolisms could be responsible for natural attenuation of aromatic compounds under sulfate-reducing and methanogenic conditions in anaerobic environments.

## Author Contributions

LZ and JT designed the research and wrote the first draft of the manuscript. ZT and JT developed the methodology and generated data. LZ, JT, and JM performed the statistical analysis. LZ, YW, and ZY contributed to the final manuscript.

## Conflict of Interest Statement

The authors declare that the research was conducted in the absence of any commercial or financial relationships that could be construed as a potential conflict of interest.
