# Supplementary material for: Enhanced Anaerobic Biodegradation of Benzoate Under Sulfate-Reducing Conditions With Conductive Iron-Oxides in Sediment of Pearl River Estuary
Source: Front Microbiol. 2019 Mar 1;10:374. doi: 10.3389/fmicb.2019.00374 (PMC6406033; doi:10.3389/fmicb.2019.00374)

# Supplementary Materials

## Enhanced Anaerobic Biodegradation of Benzoate under Sulfate-reducing Conditions with Conductive Iron-oxides in Sediment of Pearl River Estuary

**Li Zhuang[1], Ziyang Tang[2], Jinlian Ma[2], Zhen Yu[2], Yueqiang Wang[2], Jia Tang[2*]**

**Running title: Enhanced benzoate degradation under sulfate-reducing conditions by iron oxides**

Correspondence：

Jia Tang tangjia@soil.gd.cn

Table S1: Contribution of microbial Fe(III) reduction to benzoate degradation

| Treatment | feeding cycle | benozoate degraded (mM) | electrons produced from benzoate oxidation (mmol) | Fe(III) reduced (mM) | electrons consumed by Fe(III) reduction (mmol) | Electron recovery by Fe(III) reduction |
|---|---|---|---|---|---|---|
| Magnetite-amended | 1st | 2.32 | 69.6 | 7.5 | 7.5 | 11% |
| | 2nd | 2.36 | 70.8 | 1.6 | 1.6 | 2% |
| Hematite-amended | 1st | 2.31 | 69.3 | 7.0 | 7.0 | 10% |
| | 2nd | 2.46 | 73.8 | 1.6 | 1.6 | 2% |

**Figure S1** Fe(II) produced from microbial reduction of magnetite or hematite in the absence and presence of sulfate

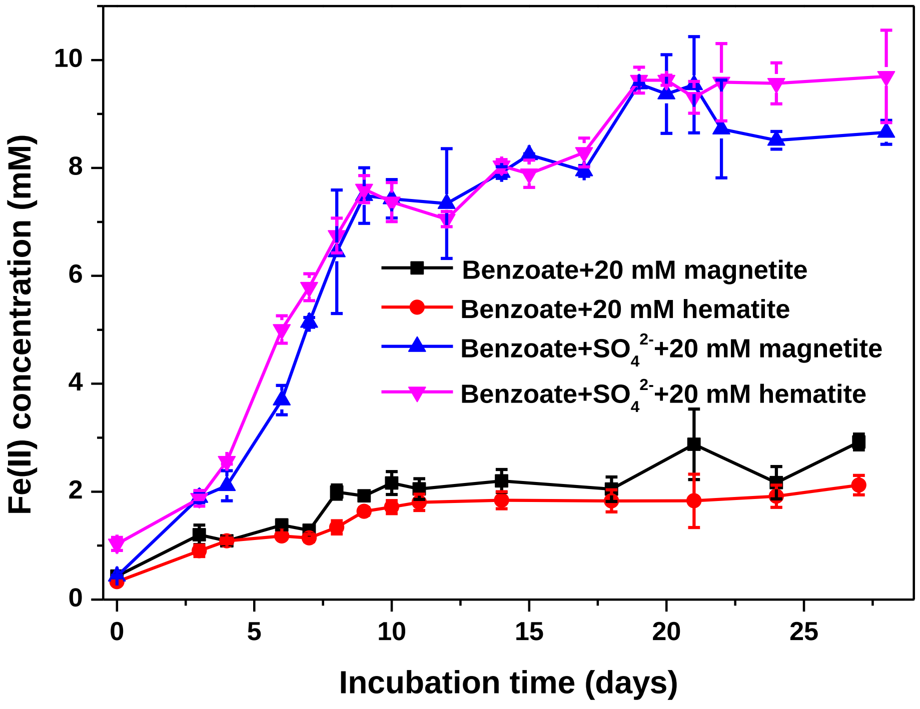

**Figure S2** A hypothetic scheme showing DIET-facilitated benzoate degradation under sulfate-reducing conditions

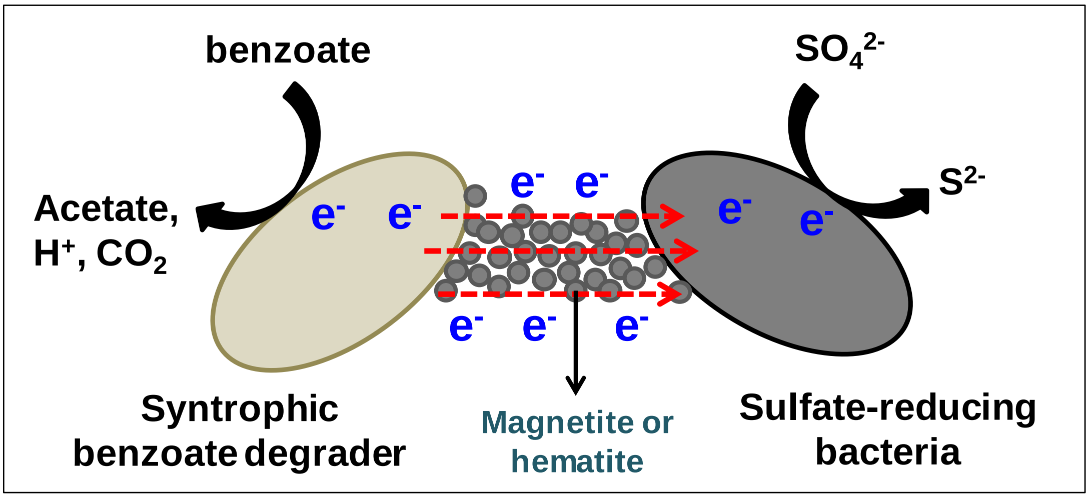


**Figure S3** A hypothetic scheme showing DIET-facilitated acetate degradation under sulfate-reducing conditions

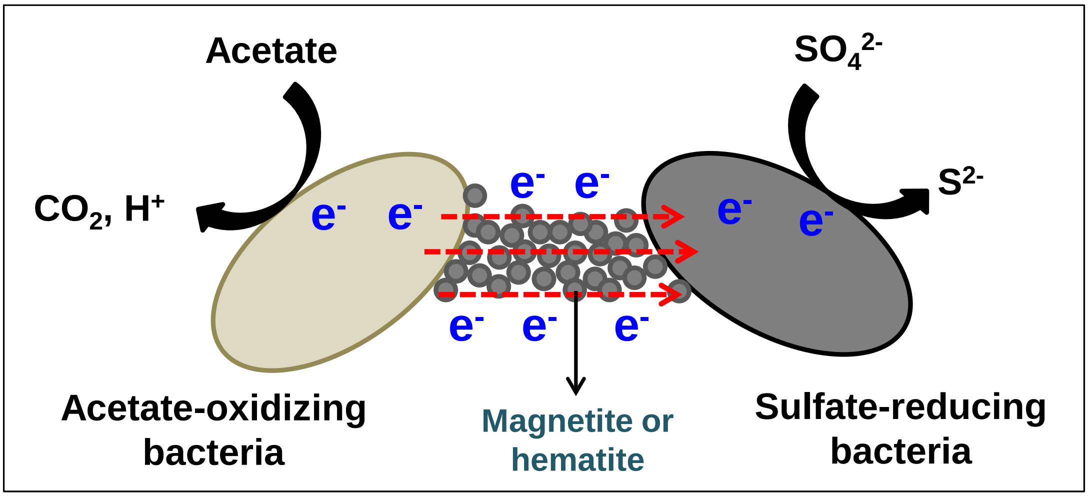

Supplement: Supplementary file 1 [file Data_Sheet_1.PDF]
